# ChASM: a statistically rigorous method for the detection of chromosomal aneuploidies in ancient DNA studies

**DOI:** 10.1093/bioinformatics/btag204

**Published:** 2026-04-28

**Authors:** Adam B Rohrlach, Jonathan Tuke, Kay Prüfer, Wolfgang Haak

**Affiliations:** Department of Archaeogenetics, Max Planck Institute for Evolutionary Anthropology, Leipzig, 04103, Germany; School of Biological Sciences, Adelaide University, Adelaide, 5005, Australia; School of Mathematical Sciences, Adelaide University, Adelaide, 5005, Australia; Department of Archaeogenetics, Max Planck Institute for Evolutionary Anthropology, Leipzig, 04103, Germany; Department of Archaeogenetics, Max Planck Institute for Evolutionary Anthropology, Leipzig, 04103, Germany

## Abstract

**Motivation:**

How individuals with conditions, disabilities or abnormalities were treated gives us valuable insights into past societies. Chromosomal aneuploidies, the presence of an abnormal number of copies of the chromosomes, represent the most common large-scale chromosomal abnormalities in human populations. Chromosomal aneuploidies can affect autosomal chromosomes (e.g. Down syndrome) as well as the sex chromosomes (e.g. Klinefelter syndrome), with physical manifestations ranging from mild to severe. While simple to identify genetically, chromosomal aneuploidies are difficult to diagnose from skeletal remains alone, as they present skeletal pathologies consistent with many other conditions.

**Results:**

Here we present *ChASM* (Chromosomal Aneuploidy Screening Methodology), a statistically rigorous Bayesian method for detecting full autosomal and sex chromosomal aneuploidies. The method leverages chromosome-wise read counts and takes into account differences in sequencing methodology, genetic coverage and condition rarity to produce posterior probability estimates for the screening of small and large databases of sequence data.

**Availability and implementation:**

To facilitate the ease of use, ChASM has been implemented in R as the package RChASM. RChASM is available under MIT license on the Comprehensive R Archive Network.

## 1 Introduction

The study of ancient DNA (aDNA) has been used to elucidate human population history and to investigate pathogens (Haak et al. 2015, Mathieson et al. 2018, Ávila-Arcos et al. 2023). An important driver of human evolution is disease and disorder, with studies investigating the history of pathogens, such as Yersinia pestis or Treponema pallidum, revealing our co-evolution (Harbeck et al. 2013, Barquera et al. 2025). Recent studies have begun to explore genetic conditions, and how these more rare and personal conditions may have been viewed in past societies (Fuchs et al. 2021, Maixner et al. 2021, Dorado-Fernández et al. 2023, Anastasiadou et al. 2024, Rohrlach et al. 2024). However, the detection of genetic conditions using aDNA is complicated as the data is often low coverage, and contamination between genetically male and female individuals can cause inconsistent genetic sex assignments (Moilanen et al. 2022).

Chromosomal aneuploidies, the presence of an abnormal number of chromosomes, are one of the most common forms of genetic abnormality in human beings, often resulting in miscarriage (Crowe et al. 1997, Orr et al. 2015). Aneuploidies can be manifest in one of three ways. Full aneuploidies, where missing or additional copies of entire chromosome(s) are present in all cells, partial aneuploidies, where only a portion of the extra chromosome(s) is present in all of the cells, and mosaic aneuploidies, where either the entire, or a portion of the extra chromosomes is present in *some* of the cells (Orr et al. 2015).

Excluding exceedingly rare cases of trisomy 22, the only aneuploidies that are not always lethal are trisomy 13 (Patau syndrome), trisomy 18 (Edwards syndrome) and trisomy 21 (Down syndrome) (Crowe et al. 1997). Symptoms frequently observed in individuals with trisomies 13 and 18, *e.g.* facial clefts, cardiac and urinary complications and limb and nervous system defects, can be severe (Springett et al. 2015), and hence the modern 5-year survival rates for trisomy 13 (9.7%) and trisomy 18 (12.3%) would have likely been much lower before the advent of modern medicine (Costello et al. 2015). Conversely, individuals today with trisomy 21 have a rate of survival to adulthood of 95% (Arumugam et al. 2016). However, trisomy 21 can lead to a wide range of developmental abnormalities, such as intellectual disabilities, cardiac defects, autoimmune disorders and recurrent infections (Carfì et al. 2014). While no individual skeletal pathology can be used to diagnose any of these trisomies, genetic diagnoses may help to explain osteological observations Rohrlach et al. (2024).

Sex chromosomal aneuploidies often lead to less life-threatening symptoms, although developmental issues are common, and some disease risk factors can be increased. For example, individuals carrying karyotype XXX are at an increased risk of malformations of the reproductive system (5–16%) (Tartaglia et al. 2010), seizures (11–15%) (Tartaglia et al. 2010) and have been observed to suffer from poor dentition (23.9%) (Wigby et al. 2020). Individuals carrying karyotype XYY have an increased risk of infertility (11.27-fold) (Berglund et al. 2020), Valgus (2527-fold) (Berglund et al. 2020) and a high observed frequency of dental issues (22%) (Bardsley et al. 2013). The most common sex chromosomal aneuploidy, XXY or Klinefelter syndrome, has been shown to lead to high rates of infertility (91–99%) (Groth et al. 2013) and an increased risk of Mediastinal cancer (500-fold) (Groth et al. 2013), as well as learning difficulties and mental health conditions (Turriff et al. 2017).

Due to the difficulty in diagnosing these conditions osteologically, very little is known about them in the archaeological record (Garcia-Heras, 2024). Several studies have uncovered cases of trisomies 18 and 21, as well as Klinefelter, Turner and Jacobs syndromes (Cassidy et al. 2020, Villalba-Mouco et al. 2021, Moilanen et al. 2022, Roca-Rada et al. 2022, Carlhoff et al. 2023, Penske et al. 2023, Rivollat et al. 2023, Anastasiadou et al. 2024, Rohrlach et al. 2024). In particular, studies by Anastasiadou et al. 2024 and Rohrlach et al. 2024 developed related but different methods that identify chromosomal aneuploidies from the distribution of mapped read counts. Anastasiadou *et al.* identify cases by calculating the coverage on chromosome 21 and the sex chromosomes, normalised by the overall autosomal coverage and applying fixed thresholds (Anastasiadou et al. 2024). Rohrlach *et. al* instead modeled read counts via a Bayesian methodology, leveraging a Dirichlet-multinomial distribution to account for differences in data generation and sample quality, but only considered autosomal trisomies.

Here we present a software package implementing a generalised approach to the Bayesian method we presented in Rohrlach et al. (2024) for detecting full chromosomal aneuploidies. We generalise the method to include sex chromosomal aneuploidies, and derive statistics to classify contamination and sequence data that diverges too far from expectation. We use simulations to show the power of the method to reliably identify cases of aneuploidies, and to calculate quality control thresholds for best use recommendations. Finally, we compare the performance of our method to that of Anastasiadou *et al.* to benchmark our method against the only currently available method for aneuploidy detection designed for aDNA.

## 2 Algorithm

### 2.1 A Distribution for read counts per autosomal chromosome

Consider the problem of describing the proportion of $N_{j}$ total reads, mapping to a set of chromosomes *A*, where |A|=n, for individual *j*. For ease of notation, let the sex chromosomes X and Y be represented as chromosomes 23 and 24, respectively.

It is tempting to assume that there exists some probability vector $p^{c}$ that describes the multinomial distribution $N_{j}∼MN\left(N_{j},p^{c}\right)$, where


$$N_{j}=\left(N_{1j},\ldots ,N_{nj}\right)$$

Is the number of reads mapping to each chromosome, and c is an *n*-dimensional vector describing the aneuploidy. We define the elements of $c=\left[c_{i}\right]$ to be the expected fold-change in reads mapping to the chromosome (or set) represented by element *i* compared to an “average non-carrier”. For example, if we consider the set of all autosomal chromosomes (n=22) to be $c_{i}=1$, then a similar c, but with $c_{21}=1.5$, would represent a karyotype with an additional copy of chromosome 21, and hence of trisomy 21 (Down syndrome). Note that the method does not attempt to diagnose partial or mosaic aneuploidies.

Unfortunately, sequencing protocols and sample quality are rarely so consistent, and hence the true distribution of $N_{j}$ is over-dispersed. To model this we also assume that $p^{c}$ has a Dirichlet prior distribution of the form $p^{c}∼\mathrm{Dirichlet}\left(\alpha \right)$. Since the Dirichlet distribution is a conjugate prior for the multinomial distribution, the posterior distribution for $N_{j}$ is a Dirichlet-multinomial (DM) distribution of the form


$$N_{j}∼DM\left(N_{j},\alpha \right).$$

We now have, for


$$\alpha_{0}=\sum_{i=1}^{n}\alpha_{i},$$

that


$$E\left[N_{ij}|\alpha \right]=N_{j}\frac{\alpha_{i}}{\alpha_{0}}$$

and


$$\text{Var}\left(N_{ij}|\alpha \right)=N_{j}\frac{\alpha_{i}}{\alpha_{0}}\left(1-\frac{\alpha_{i}}{\alpha_{0}}\right)\left(\frac{N+\alpha_{0}}{1+\alpha_{0}}\right).$$

We note that we commonly use three chromosome sets:

1. The “Autosomal set”, $A_{a}=\{1,\ldots ,22\}$. Here we consider chromosomes one through to twenty-two, and do not consider the sex chromosomes. We use this set to look at three autosomal trisomies: trisomy 13 (Patau syndrome), trisomy 18 (Edwards syndrome) and trisomy 21 (Down syndrome), but to ignore genetic sex.
2. The “Sex Chromosomal set”, $A_{s}=\left\{A_{a},23,24\right\}$. Here we consider the X and Y chromosomes, and merge all autosomal chromosomes into one set.
3. The “Autosomal Z-score set”, $A_{z}=A_{a}\setminus \{13,18,21\}$. We use this set to calculate Z-scores for assigning samples to a classification of “too unlike the reference data”. Here we again consider the autosomal chromosomes, but this time ignore chromosomes 13, 18 and 21 in order to not classify carriers of Patau, Edwards or Down syndrome as otherwise dissimilar, and again ignore genetic sex.

### 2.2 Estimating the common reference distribution

We now estimate a “common” karyotype (i.e., $c_{i}=1$ for i=1,…,n) from a quality-filtered subset of the reference data (Supplementary Section S1, available as supplementary data at *Bioinformatics* online). Of importance, we filter for (a) a lower- and upper-bound cut off of mapped reads and (b) statistical outliers when forming clusters. We then use maximum likelihood to estimate the Dirichlet parameters using the dirichlet.mle() function from the *sirt* R-package (Robitzsch and Robitzsch 2017).

From these concepts we estimate the Dirichlet distribution for the common autosomal reference, and the common references for genetically female (XX) and male (XY) individuals, denoted $\alpha^{c_{a}^{1}}$, $\alpha^{c^{XX}}$ and $\alpha^{c^{XY}}$, respectively. We use $\alpha^{c^{XY}}$ as the basis for modified sex chromosomal aneuploidies, but retain the parameter for reads mapping to the Y chromosome (denoted $\epsilon^{+}$) to account for errors in read mapping caused by, among other factors, sequencing error.

### 2.3 Adjusting α for new karyotypes

Unfortunately, especially in the cases of rare karyotypes, we likely cannot sample enough individuals with “uncommon” karyotypes in order to estimate the associated α. However, we can adjust the α for the more common karyotypes such that the expectation matches reality, and such that the variance is relatively unchanged.

Consider a karyotype where chromosome *t* undergoes a fold-change of *k*, with all others remaining the same. We then set a karyotype $c^{*}$ where $c_{i}^{*}=1$ for i≠t, but $c_{t}^{*}=k$, and the element-wise multiplication yields that


$$\alpha_{i}^{c^{*}}=\{\begin{array}{cc} \alpha_{i}, & \text{if}\;i\neq t,\\ k\alpha_{t}, & \text{if}\;i=t. \end{array}$$

for


$$\alpha_{0}^{c^{*}}=\sum_{i=1}^{n}\alpha_{i}^{c^{*}},$$

We now have that


$$\begin{array}{l} E\left[N_{i}|\alpha^{c^{*}}\right]=N_{i}\frac{\alpha_{i}^{c^{*}}}{\alpha_{0}^{c^{*}}}=\{\begin{array}{ll} N_{i}\frac{\alpha_{i}}{\alpha_{0}^{c^{*}}}, & \text{if}\;i\neq t,\\ N_{t}\frac{k\alpha_{t}}{\alpha_{0}^{c^{*}}}, & \text{if}\;i=t, \end{array}\\ =\{\begin{array}{ll} N_{j}\frac{\alpha_{i}}{\alpha_{0}}\frac{\alpha_{0}}{\alpha_{0}^{c^{*}}}, & \text{if}\;i\neq t,\\ kN_{t}\frac{\alpha_{t}}{\alpha_{0}}\frac{\alpha_{0}}{\alpha_{0}^{c^{*}}}, & \text{if}\;i=t, \end{array}\\ \approx \{\begin{array}{ll} E\left[N_{ij}|\alpha \right], & \text{if}\;i\neq t,\\ kE\left[N_{ij}|\alpha \right], & \text{if}\;i=t, \end{array} \end{array}$$

assuming that $\frac{\alpha_{0}}{\alpha_{0}^{c^{*}}}\approx 1$. This slight adjustment in expected values reflects that more (or less) reads will map to chromosome *t* as there are no more (or less) copies.

Additionally, if $\frac{\alpha_{0}}{\alpha_{0}^{c^{*}}}\approx 1$, then


$$\text{Var}\left(N_{ij}|\alpha^{c^{*}}\right)\approx \;\text{Var}\left(N_{ij}|\alpha \right).$$

Note that for sex chromosomal aneuploidies, we adjust the parameter vector for genetically male individuals, $\alpha^{c^{XY}}$, but retain the information on the proportion of reads erroneously mapping to the Y chromosome for genetically female individuals.

### 2.4 Calculating posterior probabilities

Once α has been defined, the posterior probability for an observed number of read counts per chromosome, can be calculated as


$$P\left(N_{j}|\alpha ,k\right)=\frac{\Gamma \left(\alpha_{0}\right)\Gamma \left(N_{j}+1\right)}{\Gamma \left(N_{j}+\alpha_{0}\right)}\prod_{k\in K}\frac{\Gamma \left(N_{jk}+\alpha_{k}\right)}{\Gamma \left(\alpha_{k}\right)\Gamma \left(N_{jk}+1\right)}.$$

Given the prior probability of each possible karyotype (Supplementary Section S2, available as supplementary data at *Bioinformatics* online), the posterior probability for karyotype *k* is then


(1) $$P\left(k|N_{j},\alpha \right)=\frac{P\left(N_{j}|\alpha ,k\right)P(k)}{\sum_{s\in K}P\left(N_{j}|\alpha ,ks\right)P(s)}.$$

We calculate the probabilities defined in equation 1 in the log-space using the ddirmnom() function from the *extraDistr* R-package (Wolodzko, 2020), and calculate the denominator we use the logSumExp() function from the *matrixStats* R-package (Bengtsson, 2025).

### 2.5 Identifying departure from the reference distribution

For individual *j*, assume that we have read counts for each of *n* chromosomes of the form


$$N_{j}=\left(N_{1j},\ldots ,N_{nj}\right),$$

and let the total number of reads attributed to individual *j* be


$$N_{j}=\sum_{i=1}^{n}N_{ij}.$$

Assume also that $N_{j}$ has a Dirichlet-Multinomial distribution $DM(N_{j},\alpha )$, where


$$\alpha =\left(\alpha_{1},\ldots ,\alpha_{n}\right),$$

with normalising constant


$$\alpha_{0}=\sum_{i=1}^{n}\alpha_{i},$$

that has been estimated from a reference data set (such as in 2.2).

It is known that the expected value and variance of the $N_{ij}$ are, respectively,


$$\begin{array}{c} E\left[N_{ij}\right]=N_{j}\frac{\alpha_{i}}{\alpha_{0}}\\ =\mu_{ij} \end{array}$$

and


$$\begin{array}{c} \text{Var}\left(N_{ij}\right)=N_{j}\frac{\alpha_{i}}{\alpha_{0}}\left(1-\frac{\alpha_{i}}{\alpha_{0}}\right)\left(\frac{N_{j}+\alpha_{0}}{1+\alpha_{0}}\right)\\ =\sigma_{ij}^{2}. \end{array}$$

Defining the per chromosome Z-score to be


$$Z_{ij}=\frac{N_{ij}-\mu_{ij}}{\sigma_{ij}},$$

the expected value and variance of the $Z_{ij}$ are zero and one, respectively, and assuming that $N_{ij}\gg 0$, then $Z_{ij}\overset{\cdot }{\underset{\cdot }{∼}}N(0,1)$.

Next, define


(2) $$\lambda_{j}=\sum_{i=1}^{n}Z_{ij}^{2},$$

which results in


$$\lambda_{j}\overset{\cdot }{\underset{\cdot }{∼}}\chi_{n}^{2},$$

and the number of chromosome-wise Z-scores that are considered “significant”, denoted


$$F_{j}=\sum_{i=1}^{n}\delta_{|Z_{ij}|>2}.$$

An individual is considered an outlier that does not represent the baseline if the observed $\chi^{2}$ statistic is significant, and if $F_{j}$ is sufficiently large (we suggest $F_{j}>2$).

Finally, we calculate the $\chi^{2}$ statistic on the autosomes, but without chromosomes 13, 18 and 21, resulting in the reference distribution $\chi_{19}^{2}$. We do this as, if an individual carries a (possible) trisomy on chromosomes 13 (Patau syndrome), chromosome 18 (Edwards syndrome) or chromosome 21 (Down syndrome), then we *expect* an abnormal read count on the associated chromosome.

### 2.6 Identifying when contamination may cause false XXY karyotype classification

In some cases, when there is contamination between XX and XY individuals, a mixture of the two read counts can be observed. With low numbers of reads, the variation can overlap with the distribution of reads of a true XXY carrier. For methods that only look at the ratio of reads mapping to the X and Y chromosome, this can appear like the outcome of an XXY karyotype. Since we consider the proportion of reads mapping to the autosomes, we aim to distinguish between a true case of XXY and contamination (for a sufficient total number of reads $N_{j}$).

Consider the associated Dirichlet-multinomial distributions for the XX and the XY karyotype, denoted $\alpha^{c^{XX}}$ and $\alpha^{c^{XY}}$, respectively. We assume that there is some proportion of XX-associated data, denoted γ∈[0,1], and hence the remaining proportion of 1−γ comes from XY-associated sequence data. We do not assume that there is a combination of XX or XY with any other karyotype (*i.e.* XXY or X0), and consider the probability of a random mix of two rare sex chromosomal aneuploidies negligible.

It must be then that the number of observed reads follows a distribution of the form


$$N_{j}∼DM\left(N_{j},\alpha^{c^{XX\gamma XY}}\right),$$

where


(3) $$\alpha^{c^{XX\gamma XY}}=\gamma \alpha^{c^{XX}}+(1-\gamma )\alpha^{c^{XY}}$$

We estimate γ by consider the number of reads mapping to the X and Y chromosomes, denoted $n_{j,x}$ and $n_{j,y}$, relative to the autosomes, and comparing this to the expected values,


$$E\left[N_{j}|\alpha^{c^{XX}\gamma c^{XY}}\right]=\gamma E\left[N_{j}|\alpha^{c^{XX}\gamma }\right]+(1-\gamma )E\left[N_{j}|\alpha^{c^{XY}\gamma }\right].$$

If we assume that or the reads mapping to the X and Y chromosomes are as expected, this yields that for k∈{X,Y},


$$\begin{array}{c} E\left[N_{jk}|\alpha^{c^{XX}\gamma c^{XY}}\right]=n_{js}\Rightarrow \hat{\gamma }N_{j}\frac{\alpha_{k}^{c^{XX}}}{\alpha_{0}^{c^{XX}}}+(1-\hat{\gamma })N_{j}\frac{\alpha_{k}^{c^{XY}}}{\alpha_{0}^{c^{XY}}}=n_{js}\\ \Rightarrow \hat{\gamma }N_{j}\left(\frac{\alpha_{k}^{c^{XX}}}{\alpha_{0}^{c^{XX}}}+\frac{\alpha_{k}^{c^{XY}}}{\alpha_{0}^{c^{XY}}}\right)=n_{js}-\frac{\alpha_{k}^{c^{XY}}}{\alpha_{0}^{c^{XY}}}\\ \Rightarrow \hat{\gamma }=\frac{n_{js}-\frac{\alpha_{k}^{c^{XY}}}{\alpha_{0}^{c^{XY}}}}{N_{j}\left(\frac{\alpha_{k}^{c^{XX}}}{\alpha_{0}^{c^{XX}}}+\frac{\alpha_{k}^{c^{XY}}}{\alpha_{0}^{c^{XY}}}\right)}. \end{array}$$

Using this estimate of the mixing parameter, we can now calculate $\alpha^{c^{XX\gamma XY}}$ per equation 3.

We then calculate the posterior probability of contamination, by calculating


$$P\left(C|N_{j}\right)=\frac{P\left(N_{j}|C\right)P\left(C\right)}{\sum_{k^{*}}^{K^{*}}P\left(N_{j}|k^{*}\right)},$$

where $k^{*}=\left\{XX,XY,\mathrm{XXY},C\right\}$. We restrict $K^{*}$ to only include these karyotypes as these are the only possible karyotypes that could be confused as contamination.

## 3 Results

### 3.1 Assessing the performance of ChASM via simulation

To assess the performance of ChASM, we produced $5\times 10^{5}$ simulations for each autosomal and sex chromosomal karyotype, based on empirical data, resulting in $1.1\times 10^{6}$ total simulations (see Supplementary Section S3, available as supplementary data at *Bioinformatics* online). We used these simulations to test the performance of ChASM method to correctly identify chromosomal aneuploidies, for varying levels of coverage (total number of reads).

#### 3.1.1 Simulating sex chromosomal aneuploidies

**Figure 1:**
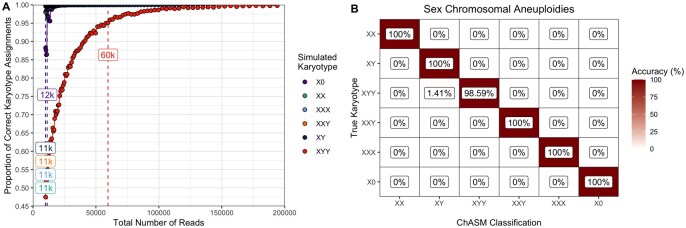
Results of the simulation study for sex chromosomal aneuploidies. (A) observed proportions of simulations that are correctly classified for each karyotype (colour) with the minimum number of total reads for which at least 95% accuracy was achieved (minimum possible 11 000 reads) as labeled boxes, (B) a confusion matrix for classification accuracy for simulations with at least 60k reads.

ChASM achieves 97.35% overall accuracy (97.33%–97.37%) when assigning sex chromosomal aneuploidies. We observe that XYY is by far the least accurately assigned karyotype with 85.24% accuracy (85.14%–85.24%), owing to the fact that the Y chromosome is significantly smaller than the X chromosome, and hence the overlap between the distribution of karyotypes XY and XYY for low read count totals is relatively large (Supplementary Figure S2, available as supplementary data at *Bioinformatics* online). From our simulations, we observe that the minimum number of total reads required to achieve 95% accuracy for all sex chromosomal aneuploidies is 60k (Fig. 1), and we apply this as the recommended minimum number of reads for analyses using ChASM.

After applying this threshold, we see the misclassification rate for karyotype XYY drop to 1.41% (1.36%–1.46%), and overall ChASM achieves 99.76% accuracy (99.76%–99.77%) and a Cohen’s κ of 0.997. Clearly, this threshold could be considered conservative for karyotypes other than XYY, and this decision can be made by researchers in context.

#### 3.1.2 Simulating autosomal aneuploidies

**Figure 2:**
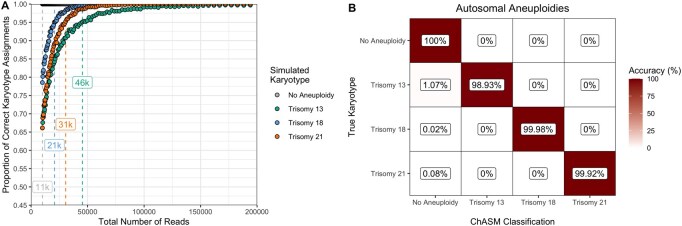
Results of the simulation study for autosomal aneuploidies. (A) observed proportions of simulations that are correctly classified for each karyotype (colour) with the minimum number of total reads for which at least 95% accuracy was achieved (minimum possible 11 000 reads) as labeled boxes, (B) a confusion matrix for classification accuracy for simulations with at least 60 000 reads.

ChASM achieves 95.16% accuracy (95.13%–95.19%) when assigning autosomal aneuploidies. Critically, we find that no simulations generated without an aneuploidy were erroneously classified as any of the possible trisomies. However, we find that all three trisomies can be misclassified at rates of between 3.51% to 9.12%, for trisomy 18 and 13, respectively (Fig. 2A). However, when we apply the minimum cut off of at least 60 000 reads, then trisomies 13, 18 and 21 are correctly classified in 98.95%, 99.98% and 99.92% of, respectively (Fig. 2B). Overall, when applying the filter, ChASM has 99.77% accuracy (99.69%–99.72%), 99.77%, and a Cohen’s κ of 0.996.

Overall, we find that ChASM reliably correctly identifies autosomal aneuploidies. Critically, the false positive rate is zero, meaning that researchers can be confident that non-common karyotype assignments are not due to model misspecification.

### 3.2 Comparison with Karyo$R_{x}R_{y}$

The only other published method for assigning chromosomal aneuploidies to aDNA is Karyo$R_{x}R_{y}$ (Anastasiadou et al. 2024). This method calculates the coverage on the X and Y chromosomes, normalised by the coverage on the autosomes, and then using coverage thresholds, identifies cases of aneuploidies. The method considers Klinefelter, Jacobs, XXX and Turner syndromes. This method also performs a similar calculation for chromosome 21 to identify trisomy 21. The thresholds for identifying aneuploidies are calculated specifically for shotgun data, but an option to change the thresholds for 1240k capture data (a commonly used enrichment method for population genetics), is also included, although the method is not calibrated for any other existing capture methods. It is due to this pre-calibrated approach that Karyo$R_{x}R_{y}$ can be run on a single individual/sample, where ChASM requires training data. Unfortunately, this means that we cannot directly run ChASM on the samples from Anastasiadou et al. (2024) as a suitable data set for training the model is currently unavailable.

Another critical difference is that ChASM uses a distributional approach to modeling read mapping. This means that ChASM can (a) return posterior probabilities of chromosomal aneuploidies, (b) take into account the prior probabilities of aneuploidies via modern estimates of the rates of prevalence, and (c) take into account coverage using the posterior Dirichlet-multinomial distribution. This approach enables ChASM to not only assign the most likely karyotype, but to also produce a measure of uncertainty around these assignments that is returned in the form of posterior probabilities and diagnostic plots.

To compare the performance of ChASM and Karyo$R_{x}R_{y}$ we performed two comparative analyses (Supplementary Section S4, available as supplementary data at *Bioinformatics* online). First, on the data for which Karyo$R_{x}R_{y}$ was calibrated to work best (shotgun and 1240k), and then on a capture assay for which Karyo$R_{x}R_{y}$ was not calibrated (immuno-capture). To compare and quantify the performance of the two methods on shotgun and 1240k sequence data, we use published data from a collection of sites from the Irish Neolithic period (Cassidy et al. 2020), the Bronze Age Iberian site of La Almoloya (ALM) (Villalba-Mouco et al. 2021), the Neolithic French site of Gurgy (GRG) (Rivollat et al. 2023), the Iron Age Thai site of Yappa Nhae (YPN) (Carlhoff et al. 2023), and the Bronze Age Bulgarian site of Yunatsite (YUN) (Penske et al. 2023). To compare and quantify the performance of the two methods on immuno-capture data, we use the published empirical data from the Neolithic site of Gurgy (Rivollat et al. 2023). We chose ALM, YPN and YUN as they have reported cases of sex chromosomal and autosomal aneuploidies. Unfortunately, we know of no such cases where aneuploidies have been identified for anything other than shotgun or 1240k capture data. However, using GRG we can still call the common genetic sexes XX and XY using both methods, and compare this to the genetic sexes assigned by Rivollat *et al.* in their study. The authors of these archaeogenetic studies report two cases of XXX syndrome (ALM062 and YPN020), one case of Klinefelter syndrome (CLL011) and two cases of trisomy 21 (YUN039 and PN07). For site and sample descriptions see Supplementary Section S5, available as supplementary data at *Bioinformatics* online.

#### 3.2.1 Shotgun and 1240k capture data

**Figure 3:**
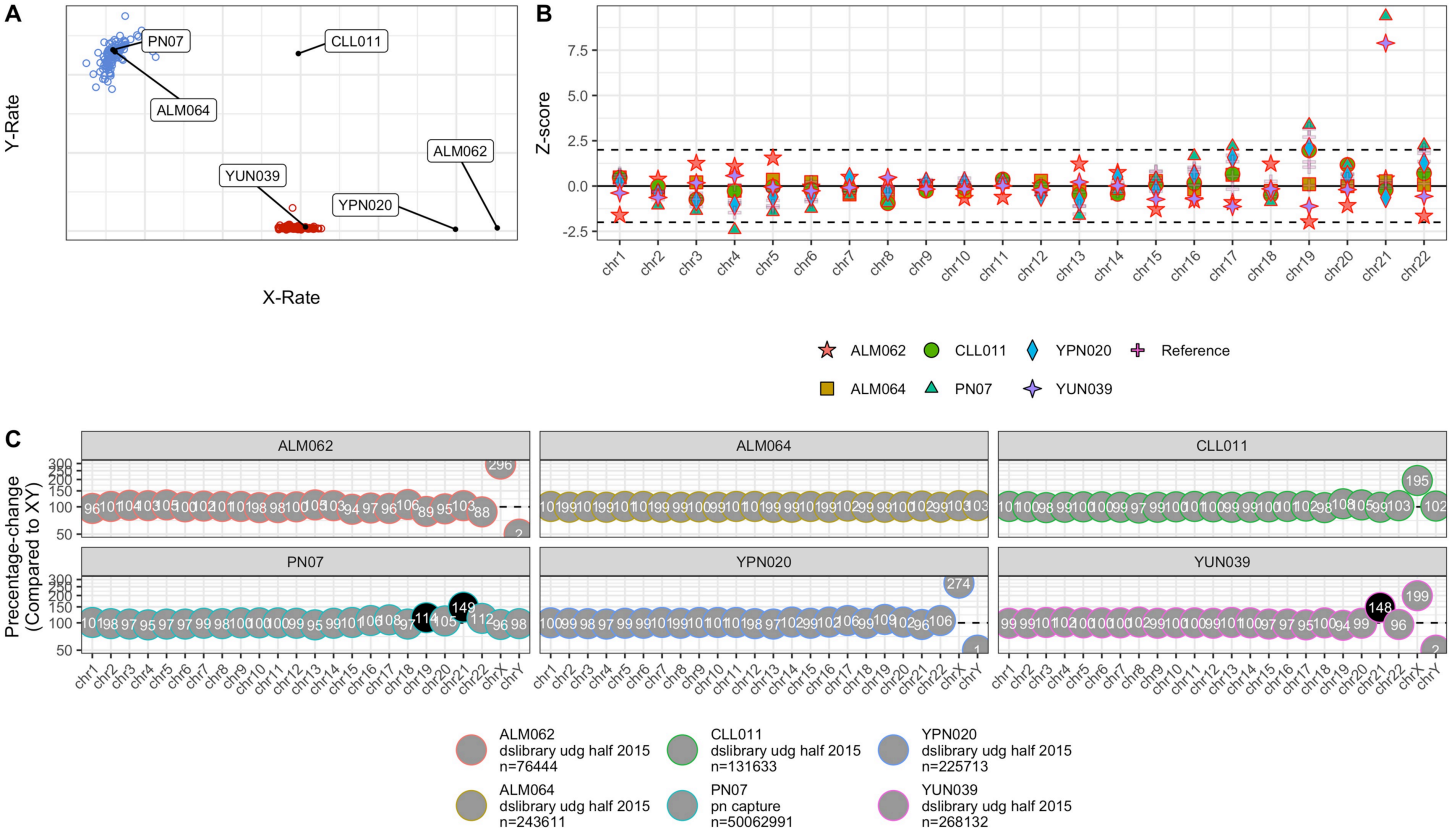
The diagnostic plot for the empirical analysis of the shotgun data for the cases of: XXX syndrome (ALM062 and YPN020), Klinefelter syndrome (CLL011), trisomy 21 (YUN039 and PN07) and an individual with no aneuploidies (ALM064). (A) a scatter plot of the proportions of reads mapping to the X and Y chromosomes, (B) Z-scores per autosomal chromosome, and (C) the percentage-increase of mapped reads per chromosomes compared to expectation (from the Dirichlet-multinomial distribution for an XY individual). Grey and black filled circles indicate |Z|≥2 for associated Z-scores.

ChASM and Karyo$R_{x}R_{y}$ correctly identify both cases of trisomy 21, the case of XXY and both cases of XXX syndrome for both shotgun and 1240k capture data. Further, we see complete agreement between the calls of XX (*n = *81) and XY (*n = *78) between the two methods. From the diagnostic plots (Fig. 3), we can see that the positions of ALM062, CLL011 and YPN020 are consistent with the reported sex chromosomal aneuploidies (Fig. 3A) and that YUN039 and PN07 yield significantly more reads to chromosome 21, consistent with the diagnoses of trisomy 21 (Fig. 3B and C). Hence, we see that ChASM and Karyo$R_{x}R_{y}$ both agree completely and correctly with the assignments of sex chromosomal and autosomal aneuploidies. Hence, we show that ChASM works equally well as Karyo$R_{x}R_{y}$ on these calibrated sequence data types.

The agreement between the methods is expected as the informative statistics used by these methods are highly correlated, with correlation coefficients of 0.999 for both $R_{x}$ and $p_{x}$ and $R_{y}$ and $p_{y}$ in our empirical examples ($p\leq 2.2\times 10^{-16}$) Hence, we expect broad agreement between the two methods for shotgun or 1240k sequence data for which careful calibration has been considered.

#### 3.2.2 Immuno-capture data

The assignments of sex chromosomal karyotypes for the immuno-capture data for GRG via ChASM completely agree with the genetic sex assignments from the authors (Rivollat et al. 2023), who used the approach given by Mittnik et al. (2016). Conversely, due to the lack of calibration to immuno-capture data, Karyo$R_{x}R_{y}$ only achieves an overall accuracy of 40.86%. This sharp decrease in accuracy is due to the fact that, while Karyo$R_{x}R_{y}$ correctly identifies every XX individual, only one of the 56 XY individuals is identified as XY, and the remaining XY individuals are instead assigned to the “Contamination” class.

**Figure 4:**
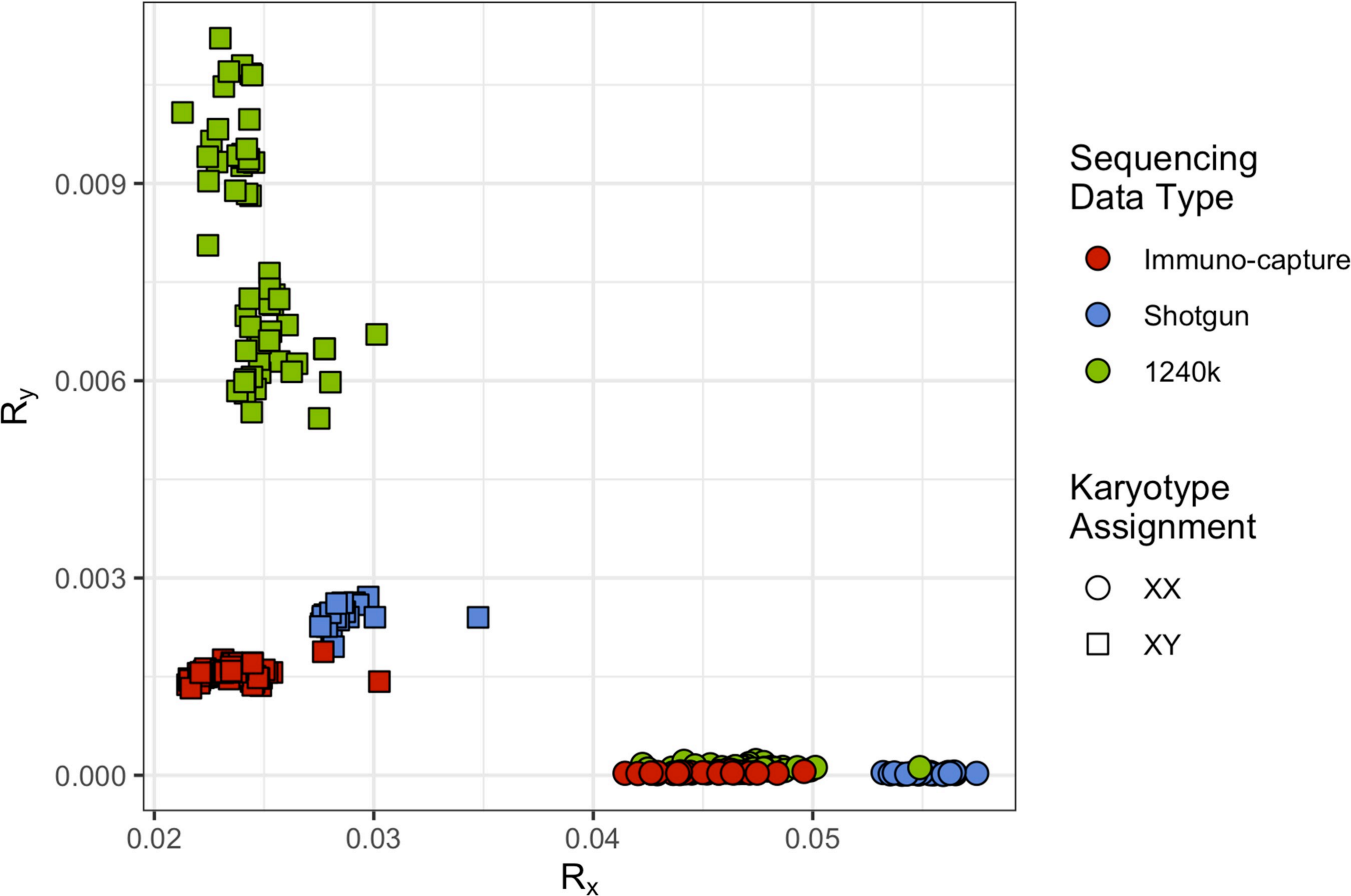
A scatter plot of the X-rate ($R_{x}$) and Y-rate ($R_{y}$) for samples from Gurgy (GRG) (Rivollat et al. 2023) as calculated by Karyo$R_{x}R_{y}$. Points are coloured by the sequencing data type, and shapes indicate the study from which the samples are sourced.

While the $R_{x}$ values are quite similar for all three sequencing data types, the $R_{y}$ values differ significantly, likely due to the desire to target informative sites on the Y chromosome in order to call Y haplogroups, and hence the increased number of sites targeted on the Y chromosome for 1240k, relative to the length of the chromosome (see Fig. 4).

It should be noted that this problem was present whether the “–capture” flag was used or not used for Karyo$R_{X}R_{y}$. For new capture assays, such as the 1.4M SNP capture assay using the Twist technology, which more than doubles the number of sites targeted on the Y chromosome from 32 670 to 81 925 (Rohland et al. 2022), this departure from calibration may present similar issues. For any new sequence data type, new thresholds could be calculated for Karyo$R_{x}R_{y}$. However, no such updates are required for ChASM as it is calibrated on training data, and is agnostic to the data generation method.

Finally, both ChASM and Karyo$R_{x}R_{y}$ identify no cases of autosomal aneuploidies, in agreement with the findings from screening the shotgun and 1240k sequence data for the same individuals.

#### 3.2.3 Departure from the reference distribution (“unusual samples”)

There are two sources of potential variability. First, capture process or biases in the DNA extraction or library preparation laboratory protocols that introduce systematic bias that can be learned from many instances of independently generated data. Second, intrinsic, sample specific features can lead to variation that is not captured by these factors but they are visible as variation across all chromosomes that are unusual compared to other datasets of the same make. An extreme example of this could be accidentally including a sample which was produced using a capture assay in an analysis of samples produced using shotgun data.

In such cases, it is possible that samples do not resemble any of the possible α associated with a karyotype. Under these conditions, one of the karyotypes will fit “best”, but we must also assess that they fit sufficiently well. To identify departures from the reference distribution, we calculate λ, a $\chi^{2}$-statistic (Section 2.5). To assess the performance of λ, and based on empirical data, we simulated $10^{5}$ realisations of read counts, across varying levels of total read counts, half of which are made to depart from the reference distribution (Supplementary Section S6, available as supplementary data at *Bioinformatics* online). As the amount of “departure” is a continuous spectrum also, *i.e.* some samples are more abnormal than other, we varied how much we modified α (defined as the “error factor”).

**Figure 5:**
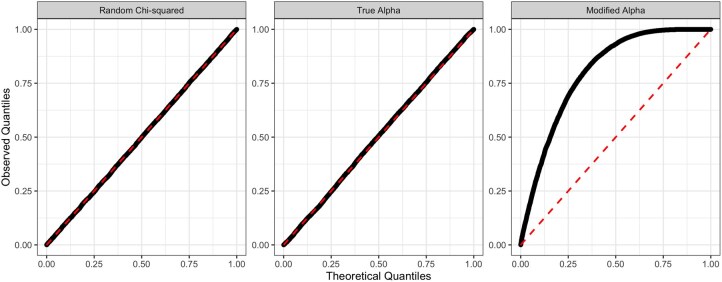
QQ-plots for the random sample from a $\chi_{22}^{2}$ distribution, from the simulations with the true α, and with a modified α vs the theoretical quantiles from a $\chi_{22}^{2}$ distribution.

We see that the test statistics produced under the modified simulations do not fall on the red dashed line, indicating a poor fit to the $\chi_{22}^{2}$ reference distribution. However, the test statistics calculated from the true simulations fit the theoretical quantiles, as well as the random sample from the true reference distribution (Fig. 5). Hence, our reference distribution for $\lambda_{j}$ appears to be well calibrated.

We were then interested in the performance of the method in predicting whether a realisation was produced from a true or a modified simulation. We observe that the $\lambda_{j}$ are significantly ($p<2.2\times 10^{-16}$) greater for modified simulations (Supplementary Figure 1A, available as supplementary data at *Bioinformatics* online). We also compared the associated p-values for the tests, and note that while the p-values from the true simulations appear to follow the expected uniform distribution, the p-values from the modified simulations are highly left-skewed (Supplementary Section S7, Supplementary Figure 1B, available as supplementary data at *Bioinformatics* online).

To test the effect of simulation parameters on λ, we modeled the test outcomes using logistic regression (Supplementary Section S6, available as supplementary data at *Bioinformatics* online). We find that for simulations where α is unmodified, total read count and error factor have no significant effect on the performance of the test statistic λ (p=0.069 and p=0.177, respectively), and yields an expected false positive rate of 5%. For the simulations where α was modified, both read count and error factor were significant ($2\times 10^{-16}$), and the ability of λ to correctly identify unusual samples increase as the total read count and the error factor increase, with the effect size of the error factor 3.41 times larger than the total read count. Finally, we find that the distribution of the read proportions need only have an error factor of around 3% for λ to have an expected accuracy of 95% or greater of detecting “unusual samples” (Supplementary Figure 1C, available as supplementary data at *Bioinformatics* online).

#### 3.2.4 Contamination

In order to test the usefulness of our method for detecting contamination (of karyotypes XY into XX), we simulated 250 000 realisations without contamination, and 250 000 realisations with contamination rates between 5% and 95% (Supplementary Section S8, available as supplementary data at *Bioinformatics* online). We find that our method has an overall accuracy of only 92.06%. While the sensitivity is high (99.82%), the specificity is lower (86.41%), indicating that the false positive rate would be too high for ChASM to be used as a generalised method for contamination.

However, we only aim to use the contamination estimates from ChASM to estimate if contamination may drive the classification of a sample as karyotype XXY. For the 52 218 simulations of contamination that resulted in a classification of XXY, the simulated contamination rate was between 14.45% XX into XY, or 28.23% XY into XX (Supplementary Section S9, Supplementary Figure S2, available as supplementary data at *Bioinformatics* online). In these cases, only 5/52218 simulations ($9.58\times 10^{-3}$%) failed to identify that contamination was the true reason for the misclassification for samples with at least 60 000 reads, resulting in 99.9% accuracy. The method still performs well for low-coverage samples, with 99.1% accuracy achieved for samples with between 10 000 and 60 000 reads. Hence, if a sample results in a call of XXY, then the rate of contamination is likely to be quite high, and the contamination warning generated by ChASM will be reliable, even for extremely low-coverage samples. Nevertheless, we suggest using established methods to detect contamination (Olalde et al. 2014, Renaud et al. 2015, Peyrégne and Peter, 2020) that should be implemented before any downstream aDNA analysis, and that this method should not be used for general contamination estimates.

#### 3.2.5 Computational performance of RChASM

We explored the runtime and memory usage of performing a full analysis in RChASM, and how this will scale to larger and larger datasets (Supplementary Section S10, available as supplementary data at *Bioinformatics* online). On average, bam files take approximately 7.04 s per bam file. However, we point out that bam files only need to be processed once, and we then encourage users to keep a record of these process read counts to add to as new data is produced. The time to run RChASM grows linearly with the number of samples in the analysis ($r^{1}\approx 1$). We found that an additional $9.09\times 10^{-4}$ s are required per sample (95% CI $\left[9.01\times 10^{-4},9.17\times 10^{-4}\right]$), indicating that an analysis of 100 000 samples would require approximately between 90.2 and 91.7 s. The maximum amount of memory required for RChASM did not grow linearly with the number of samples, and instead appears to plateau at around 1305 MB of memory. For integration into workflows, we report that we use no unusual dependencies and we employ widely-used standard software (samtools, bedtools, R, bash, perl).

## 4 Discussion

ChASM can be used in single studies where the statistically required minimum of n=23 samples with identical data production approach is met. However, the more samples of a similar data production type that can be utilised, the better the Dirichlet-multinomial distribution will be calibrated. Thus, we encourage researchers to build large legacy databases of read counts for regular screening of their data, which could form part of an analysis pipeline. Since ChASM requires only 60 000 reads mapping to the human genome, regardless of the data sequencing type, large databases of read counts can be generated from shotgun screening data. However, we encourage researchers to carefully consider only combining data that has been generated and processed under similar conditions and thresholds, which can negatively affect the underlying model, and for which the effects will likely be more noticeable for smaller data sets.

ChASM is a powerful and statistically rigorous tool for detecting chromosomal aneuploidies. We show that the method works for samples with as low as 60 000 mapped reads, equating to approximately 0.001X (assuming a mean read length of 45 bp (Renaud et al. 2014)) coverage. However, we note that as we identified only five known aneuploidies from published data, the robustness of the method to real-world artifacts in the data may reduce empirical accuracy in some cases. Additionally, we show that the diagnostic statistic used by ChASM to detect departures from the Dirichlet-multinomial distribution performs well. We also suggest that calculating the proportion of samples that deviate from the reference distribution from sequencing runs may perform well as a quality control tool to detect abnormal sequencing runs. Finally, we show that ChASM can reliably detect levels of contamination where they may cause spurious classifications of XXY. However, we warn that this method of contamination estimation lacks power to detect low amounts of contamination, and hence ChASM should not be used as the sole method for detecting contamination in studies.

While cases of chromosomal aneuploidies are not frequent, they still represent the most common class of large-scale chromosomal abnormalities. As the number of available ancient genomes continues to rapidly increase, screening for aneuploidies can offer potential explanations for skeletal abnormalities or pathologies. Autosomal aneuploidies can lead to significant health challenges in individuals (Orr et al. 2015), for which no osteological markers are diagnostic. Sex chromosomal aneuploidies, while often undiagnosed in modern individuals, have been shown to lead to symptoms which may have caused health issues, mental developmental issues or elevated rates of gender dysphoria (Soibi-Harry et al. 2025). Detecting and diagnosing cases of aneuploidies leads to a more complete understanding of an individual, and the community in which they lived, as well as osteological markers that may otherwise have many other possible explanations.

## Supplementary Material

btag204_Supplementary_Data

## Data Availability

We implemented ChASM in the R-package RChASM, available on The Comprehensive R Archive Network (CRAN). A comprehensive vignette for each step of a standard analysis is available at https://jonotuke.github.io/RChASM/articles/example_analysis.html. All scripts for the R analyses presented in the study can be found at https://github.com/BenRohrlach/ChASM_RAnalyses and read counts for the simulated and empirical data are available at https://zenodo.org/records/18657465. For studies where the minimum threshold of n=23 is not met, we make available a data set of published read counts (ChASM_empirical.tsv) to which researchers can append their read counts, but urge caution in interpreting the results as the reference data may not perfectly match the additional user-data.
