# Supplementary material for: ChASM: a statistically rigorous method for the detection of chromosomal aneuploidies in ancient DNA studies
Source: Bioinformatics. 2026 Apr 28;42(5):btag204. doi: 10.1093/bioinformatics/btag204 (PMC13198388; doi:10.1093/bioinformatics/btag204)
Supplement: btag204_Supplementary_Data [file btag204_supplementary_data.pdf]

ChASM: A Statistically Rigorous Method for the Detection of Chromosomal Aneuploidies in Ancient DNA Studies
Supplementary Notes

Adam B. Rohrlach
Department of Archaeogenetics
Max Planck Institute for Evolutionary Anthropology
Leipzig, Germany
adam_ben_rohrlachch@eva.mpg.de

Jonathan Tuke
School of Mathematical Sciences
Adelaide University
Adelaide, Australia
simon.tuke@adelaide.edu.au

Kay Prüfer
Department of Archaeogenetics
Max Planck Institute for Evolutionary Anthropology
Leipzig, Germany
pruefer@eva.mpg.de

Wolfgang Haak
Department of Archaeogenetics
Max Planck Institute for Evolutionary Anthropology
Leipzig, Germany
wolfgang_haak@eva.mpg.de

S1 Selecting the reference set for parameter estimation

When estimating the prior distribution from the reference data, we filter the data for some characteristics. Note, that we can still use this distribution to calculate probabilities on data that do not meet these thresholds, but use a filtered data set for estimating the Dirichlet parameters, allowing the posterior Dirichlet-multinomial to account for the additional noise caused by sample quality.

For all three common chromosomes sets (A_a, A_s and A_z) , we set a lower (N_{min}) and an upper (N_{max}) threshold for the total number of mapped reads. However, for calculating the prior distributions for sex chromosomal aneuploidies, we must consider that there are two common karyotypes, XX and YY. To estimate these, we first cluster the high-coverage filtered data into three groups using their coordinates in three-dimensional space defined by p_{jx} and p_{jy} where

$$p_{js} = \frac{N_{j,s}}{N_j}, \;\; s \in \{A_a, 23, 24\} \,,$$

are the proportion of reads mapping to the X and Y chromosomes, respectively, for individual j. Since these are proportions of reads, with enough reads to assume that a three-dimensional Gaussian distribution is asymptotically reasonable, we use hierarchical model-based agglomerative clustering, estimated using the EM-algorithm, as implemented in the *mclust* package for R[Scrucca et al., 2023]. This produces three distinct clusters: one associated with the XX and XY karyotypes, and a third "noise" cluster.

From these quality-control filtered and clustered data sets, we estimate the parameters for the Dirichlet prior distribution.

S2 Prior probabilities for karyotypes

We use modern estimates of the frequency of the XXY, XYY, X0 and XXX karyotypes for prior probabilities of observing each karyotype[cite].

Supplementary Table 1: Karyotypes of interest for this method, the associated adjustment vectors and prevalence.

Karyotype	Name	$\boldsymbol{c}$	Prevalence
T13	Patau syndrome	$\boldsymbol{c}_a^{T_{13}} = [\delta_{i=13}]_{i \in \boldsymbol{A}_a}$	1:7,143 births[Mai et al., 2013]
T18	Edwards syndrome	$\boldsymbol{c}_a^{T_{18}} = [\delta_{i=18}]_{i \in \boldsymbol{A}_a}$	1:3,226 births[Mai et al., 2013]
T21	Down syndrome	$\boldsymbol{c}_a^{T_{21}} = [\delta_{i=21}]_{i \in \boldsymbol{A}_a}$	1:705 births[Mai et al., 2013]
XXY	Klinefelter syndrome	$(2, 1, 1)$	1:750 male births[Skuse et al., 2018]
XYY	Jacobs syndrome	$(1, 2, 1)$	1:1,000 male births[Skuse et al., 2018]
XXX	Trisomy X	$(3, \epsilon^+, 1)$	1:1,000 female births[Skuse et al., 2018]
X0	Turner syndrome	$(1, \epsilon^+, 1)$	1:2,500 male births[Skuse et al., 2018]

We then define the prior probabilities of XX and XY to be the remaining probability, but weighted by the ratio of sex bias[Chao et al., 2019], *i.e.*,

$$P\left(XY\right) = \frac{105}{205}\left[1 - \sum_{k \in K} P(K)\right],$$

and

$$P\left(XX\right) = \frac{100}{205}\left[1 - \sum_{k \in K} P(K)\right],$$

where $K = \{XXY, XYY, X0, XXX\}$.

S3 Simulations for the accuracy of the method to detect aneuploidies

To test the utility of the method to detect autosomal and sex chromosomal aneuploidies, and to find a minimum number of mapped reads for reliable results from the method, we performed two simulation studies: one for sex chromosomal aneuploidies, and one for autosomal aneuploidies. We aimed to avoid a cumbersome simulation study where we merge sequence data such that additional reads mapping to chromosomes of interest are artificially inflated (according to observed read counts), and then calculate read counts. We avoid this by directly inflating read counts calculated on observed data, and by randomly down-sampling these data to explore the effect of coverage in the following way.

To represent realistic data, we used (resampled with replacement) empirical shotgun data, based on empirical read counts from the published data (Section 5) filtered such that there were between 10^5 and 10^8 total mapped reads for an individual, and removing the individuals identified as having aneuploidies, in the following way. This resulted in 122 different individuals. We defined a grid of values of the total number of reads, $N_T \in \left[10^3, 10^5\right]$, and a number of repetitions per total read count, $N_r = 5 \times 10^3$, resulting in a total of 5×10^5 realisations per aneuploidy type.

Then for each realisation, we randomly sample the read counts for an observed individual, normalise these counts as proportions, and sample a simulated number of reads according to the value of N_T. We then take this simulated read count vector and for each aneuploidy, calculate the number of reads to add or subtract to the associated chromosome(s) from a Gaussian distribution of the form

$$N \sim \left(\frac{n}{2\delta_a}, \frac{n}{\delta_a 200}\right),$$

where n is the number of observed reads in the associated chromosome, and $\delta_a = 1/2$ if the aneuploidy is autosomal. Then for all realisations, we called karyotypes using the Dirichlet-multinomial distributions estimated on just the 122 individuals. We recorded whether the karyotype was called correctly, and recorded the proportion of correct karyotype calls per total number of reads.

S4 Statistical tests for assessing performance

All analyses and plots are implemented using the R statistical software package[R Core Team, 2025].To assess and compare the performance of the methods, we calculate confusion matrices as implemented in the *caret* package[Kuhn, 2008]. Logistic regression models were fit using the `glm()` function from the *stats* package[R Core Team, 2025]. All plots were produced using the *ggplot2*[Wickham, 2016].

S5 Empirical data descriptions

All internal data (all data except from Cassidy *et al.* 2020) was produced using the dsLibrary UDG half 2015 protocol, generated at the Max Planck Institute for the Science of Human History in Jena, Germany, and then the Max Planck Institute for Evolutionary Anthropology in Leipzig, Germany[Yates et al., 2020]. After adapter trimming using leeHom, reads were aligned to the human reference genome GRCh37 using BWA (version: 0.7.12; parameters: -n 0.01 -o 2 -l 16500)[Li, 2013, Renaud et al., 2014]. Sequence reads were filtered for a minimum sequence length of 35 and a mapping quality of at least 25.

S5.1 Carlhoff *et al.* 2023: Yappa Nhae 2 Cave (YPN)

Yappa Nhae 2 Cave (YPN) is a cave located in the Mae La Na basin near Ya-Pa Nae village in Northwestern Thailand. Four log coffins were discovered in the middle, upper and lower chamber indirectly dated to 70 BCE to 750 CE. The sequence data is comprised of n=19 shotgun samples and n=31 1240k samples. For a full description of the site and individuals, see Carlhoff *et al.* 2023[Carlhoff et al., 2023].

S5.2 Cassidy *et al.* 2020

For the purpose of analysing sequence data that was not generated at the Max Planck Institute for Human History, or the Max Planck Institute for Evolutionary Anthropology, we include a sub-sample of the individuals from Cassidy *et al.* 2020[Cassidy et al., 2020]. These individuals come from a collection of cave and monumental sites from Mesolithic to Late Neolithic Ireland. The sequence data is comprised of n=46 shotgun samples. For a full list of the samples and individuals that were analysed, see Supplementary Table SXX[Cassidy et al., 2020].

S5.3 Penske *et al.* 2023: Tell Yunatsite (YUN)

Tell Yunatsite is among the largest tell settlements in Bulgaria. The site is situated in southern Bulgaria, around 1 km southwest of the village of Yunatsite. Tell Yunatsite comprises cemeteries dating from the Medieval to the Thracian period settlement, although the samples we analyse here data to the Bronze Age (3rd millennium BCE) and Chalcolithic layers (5th millennium BCE). For a full description of the site and individuals, see Penske *et al* 2023[Penske et al., 2023].

S5.4 Rivollat *et al.* 2023: Gurgy 'les Noisats' (GRG)

Gurgy 'les Noisats' (GRG) is a Neolithic burial site in the Cerny cultural horizon, located in Auxerrois, on the Paris basin. Gurgy dates to the fifth millennium BCE, and while it is located close to many monumental sites, contains no monumental architecture of its own. Further, due to the different body positions, architectural variation from various cultural influences, and a lack of burial goods, a direct association to the Cerny culture is difficult. The sequence data is represented by n=53 shotgun samples, n=107 1240k samples and n=93 immuno-capture samples. For a full description of the site and individuals, see Rivollat *et al* 2023[Rivollat et al., 2023].

S5.5 Villabla *et al.* 2021: La Almoloya (ALM) and Cueva de las Lechuzas (CLL)

La Almoloya (ALM) is a large Bronze Age settlement, associated with the El Agar, and located in the northern foothills of Sierra Espuña, in South Eastern Iberia. It sits on a plateau around 585 metres above sea level, and was continuously occupied from 2200 to 1550 cal BCE, spanning the entire El Agar period.

Cueva de las Lechuzas (CLL) is a cave with burials dating to the Late Neolithic/Chalcolithic period, located on the eastern slope of a small in the centre of the Villena basin, in Alicante, South Eastern Iberia. This collective burial contained burial goods directly dating the burials to 3300 to 2300 cal BCE.

The sequence is comprised of n=56 shotgun samples and n=82 1240k samples. For a full description of the site and individuals, see Villalba-Mouco *et al.* 2021[Villalba-Mouco et al., 2021].

S5.6 Karyotype assignments

S6 Simulations for the performance of λ, the χ^2 test-statistic

To test the performance of the statistic suggested in Section 2.5 of the main text, we performed a simulation study. We began by using the parameters from a DM estimated from our shotgun data, denoted $\boldsymbol{\alpha}$. We simulated 10^5 realisations,

	XX	XY	XYY	XXY	XXX	X	Total
Carlhoff 2023	8	10	0	0	1	0	19
Cassidy 2020	8	38	0	0	0	0	46
Penske 2023	22	8	0	0	0	0	30
Rivollat 2023	20	33	0	0	0	0	53
Villalba 2021	27	27	0	1	1	0	56
Total	85	116	0	1	2	0	204

Supplementary Table 2: The number of assignments to each sex chromosomal karyotype for the shotgun sequencing data. Data is filtered such that the total number of reads is at least 60,000. Individuals may appear more than once if sequenced more than once.

	XX	XY	XYY	XXY	XXX	X	Total
Carlhoff 2023	15	15	0	0	1	0	31
Penske 2023	28	9	0	0	0	0	37
Rivollat 2023	41	66	0	0	0	0	107
Villalba 2021	45	35	0	1	1	0	82
Total	129	125	0	1	2	0	257

Supplementary Table 3: The number of assignments to each sex chromosomal karyotype for the 1240k capture sequencing data. Data is filtered such that the total number of reads is at least 60,000. Individuals may appear more than once if sequenced more than once.

	XX	XY	XYY	XXY	XXX	X	Total
Rivollat 2023	37	56	0	0	0	0	93

Supplementary Table 4: The number of assignments to each sex chromosomal karyotype for the immuno-capture sequencing data. Data is filtered such that the total number of reads is at least 60,000. Individuals may appear more than once if sequenced more than once.

under using either $\boldsymbol{\alpha}$ (the "true simulations") or a modified form of $\boldsymbol{\alpha}$ (the "modified simulations"). For each simulation we:

- used $\boldsymbol{\alpha}$ or a modified $\boldsymbol{\alpha}$, with equal probability,
- sampled a total number of reads $N_j \sim \mathrm{U}\left(10^4, 10^5\right)$,
- sampled a "modification factor" $q_{ij} \sim \mathrm{N}\left(\mu_i, \sigma_i^2\right)$ for each chromosome $i \in \{1, \cdots, n\}$ (w)e obtained the μ_i and σ_i^2 by calculating the proportional increase in observed read counts above and below expectation in the empirical data), and
- sampled a number of chromosome that are affected, F_j, which is sampled from the empirical data (w)e obtained this distribution by calculating the number of "flags" for the observed samples for which the observed p-value was less than 0.05).

We simulated F as we did not want to overestimate the performance of the method due to unrealistic simulations where *everything* is affected. Hence, if we were simulating a modified simulation, then the non-normalised modified DM parameters are

$$\begin{aligned}\boldsymbol{\alpha}'_j &= [\alpha_i + \alpha_i q_{ij} \Delta_{ij}] \\ &= \left[\alpha'_{ij}\right],\end{aligned}$$

where, for $\boldsymbol{\theta}_j$, a random sample (without replacement) of size F_j from $\{1, \cdots, n\}$, Δ_{ij} equals one if $i \in \boldsymbol{\theta}_j$, and is zero otherwise. We then normalise this vector and use the modified alpha of the form

$$\boldsymbol{\alpha}_j^M = \left[\frac{\alpha'_{ij}}{\sum_{k=1}^{n} \alpha'_{kj}}\right].$$

We then calculated the associated test statistics as defined in Equation 2 of the main text, and for each realisation recorded the mean of the absolute value of the non-zero modification factors ($\bar{q}_j$) and the number of total reads (N_j), and produced QQ-plots for the observed values of the λ_j calculated from the simulation study, and the random sample of 10,000 values from a χ^2_{22} distribution using the *rchisq()* function in R (Figure 5).

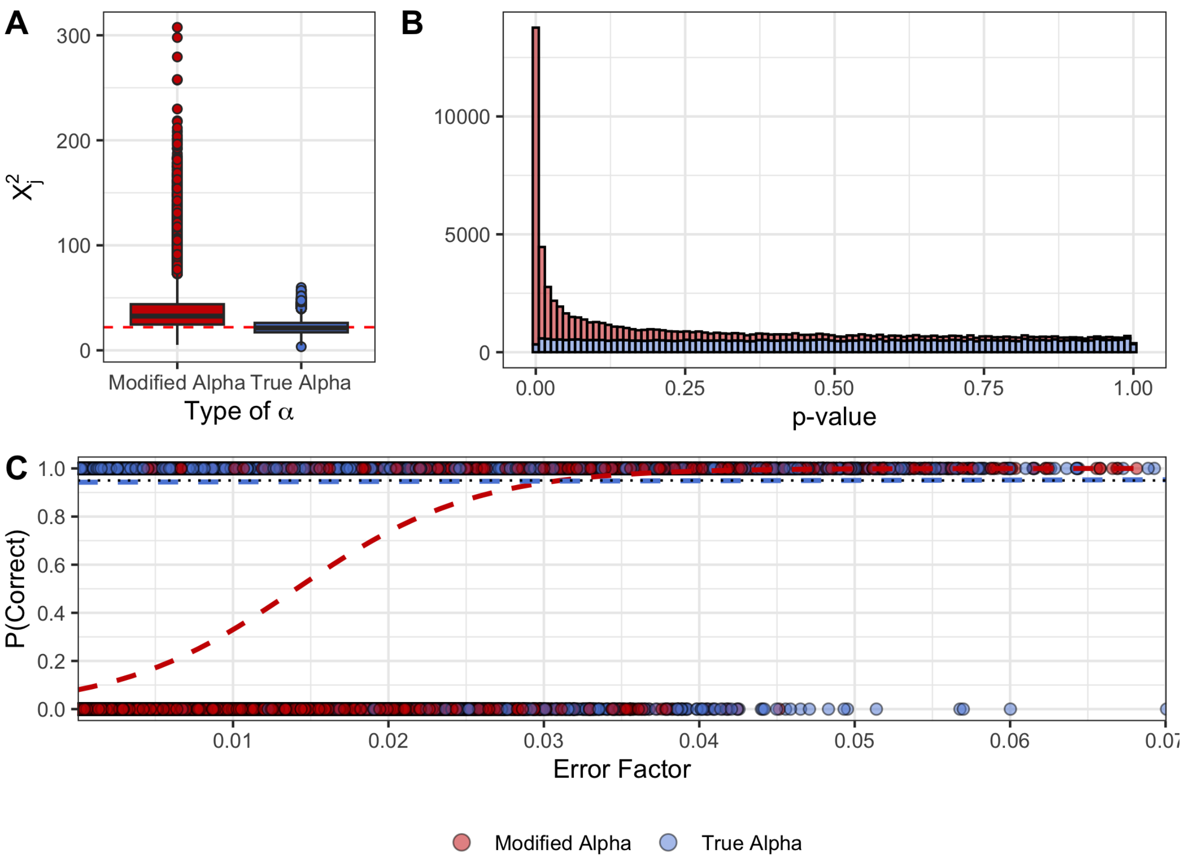

Supplementary Figure 1: (A) boxplot of the λ_j calculated from the simulations under the true and modified Dirichlet-multinomial distributions, (B) a histogram of the associated p-values calculated from the observed test-statistics, and (C) a logistic regression of correctly predicting the outcome of a simulation as either from the true or a modified $\boldsymbol{\alpha}$ against the average absolute value of the non-zero modification factors, $\bar{q}_j$.

S7 Logistic regression of the performance of λ, the χ^2 test-statistic

To explore the robustness of the test statistic (for simulations with total read count greater than 60,000) with respect to the simulation parameters, we fit a logistic regression of the form

$$\log\left(\frac{p}{1-p}\right) = \beta_0 + \beta_1 d_j + \beta_2 \bar{q} + \beta_3 d_j \bar{q} + \beta_4 n_j + \beta_5 d_j n_j,$$

where d_j is an indicator variable such that it is zero if the realisation came from a modified simulation, and is one otherwise, $\bar{q}_j$ is the mean, absolute, non-zero modification factor, and n_j is the total number of simulated reads. We find that this model cannot be simplified ($p < 2.2 \times 10^{-16}$).

The estimated coefficients for β_2 and β_4 are not significantly different from zero ($p = 0.522$ and $p = 0.921$, respectively), indicating that for the true simulations, the total read count and error rate have no significant effect on the performance of the test statistic λ. In fact, the predicted probability of correctly identifying these simulations is approximately 0.95, in line with expectation at the 5% significance level.

However, we find that both read count and error rate are significant predictors for the predictive power for the modified simulations. While an increase in the total number of reads improved the performance of λ (effect size 1.31), the error rate was a far more impactful variable, with performance increasing the more $\boldsymbol{\alpha}_j^M$ deviated from $\boldsymbol{\alpha}$ (effect size 5.2). Overall, we observe that the probability of identifying true positives first exceeds a probability of 0.95 after $\boldsymbol{\alpha}_j^M$ deviates from $\boldsymbol{\alpha}$ by an average of approximately 3% (Figure 1C), which is less the observed mean deviation for all chromosomes, except for chromosome one (2.86%).

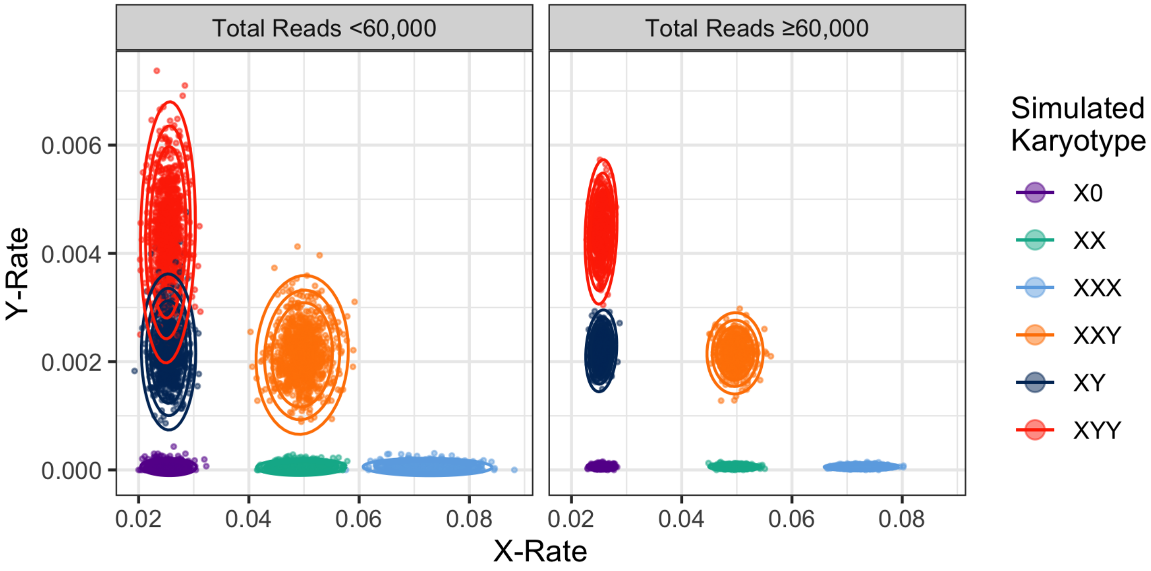

Supplementary Figure 2: Simulated X-rate (x-axis) and Y-Rate (y-axis) for the possible sex chromosomal aneuploidies (colour), faceted by the total number of reads being less than 60k (left panel) or greater than 60k (right panel). We take a random sample of 10,000 points to make visualisations easier, and include 95%, 99% and 99.9% confidence ellipses.

S8 Misclassification of XYY as XY

We find that for low read count totals ($\leq$ 60k), XYY is misclassified as XY at a rate of 23.64%. The reason for this can be seen when plotting the simulated X-rate (p_x) and Y-rate ()p_y), considering that the Y chromosome is much smaller than X chromosome and that the prior probability of XY is much greater than for XYY (see Table 1). Since p_y is quite small, $2p_y$ is also quite small, and if the read counts are low, then the variability of p_y is high relative to the expected value. Hence the distributions of XYY and XY overlap significantly (Figure 2). Note that due to the prior probabilities of XYY and XY , we rarely see XY misclassified as XYY.

S9 Required amount of contamination for XXY classification

To explore the amount of contamination required for ChASM to erroneously call a contaminated sample as XXY, we calculated the minimum and maximum values of the contamination rate γ where this occurred. We then also calculated the accuracy of ChASM to identify contamination for values of γ discretised into bins of width 1%, *i.e.* 5%, 6%,...,95%.

S10 Runtime and memory usage benchmarking

To explore the runtime and memory usage of running a full analysis in RChASM we used the Linux `time` command to benchmark the time taken to process bam files, and the `microbenchmark` package for RMersmann [2024]. For microbenchmark, we used 5 random resamples per number of samples To test these metrics at different scales of analysis, we randomly sampled the bam files from the empirical data used in Section 3.2, with replacement, from 500 to 15,000 samples, in steps of 500 samples.

We used the *lm()* function[R Core Team, 2025] to fit the no-intercept linear regression of sample size against runtime for RChASM, and fit an adjusted logit curve to the maximum memory usage against sample size using the *nlsLM()* function from the `minpack.lm` R package[Elzhov et al., 2016]. All tests were performed on an Apple M3 Pro with 18 GB of RAM.

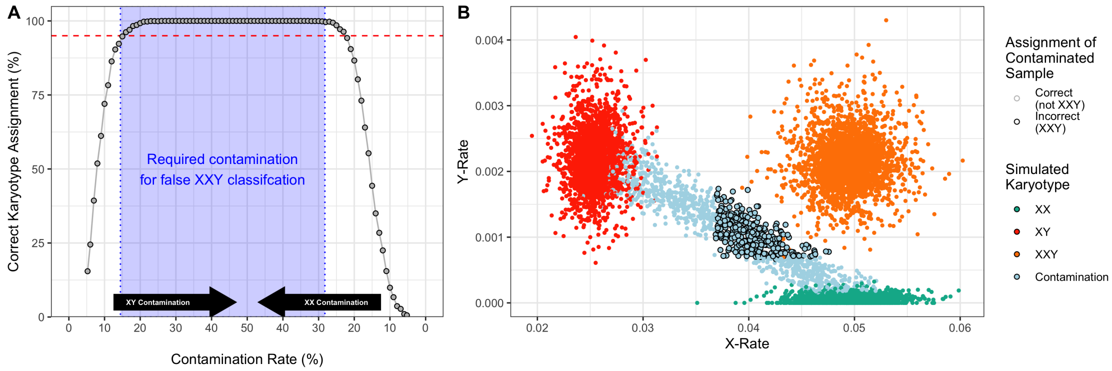

Supplementary Figure 3: (A) The percentage of correctly identified simulations with contamination (y-axis) against the amount of simulated contamination (x-axis), binned into bins of width one percentage point. The red dashed line indicates 95% accuracy, and the blue region indicates levels of contamination that resulted in a karyotype classification of XXY. (B) a scatter plot of the x- and y-rates for simulated karyotypes XX (green), XY (red), XXY (orange) and XX contaminated with XY reads (light blue), where bold symbols indicate samples that are incorrectly assigned as XXY by ChASM.

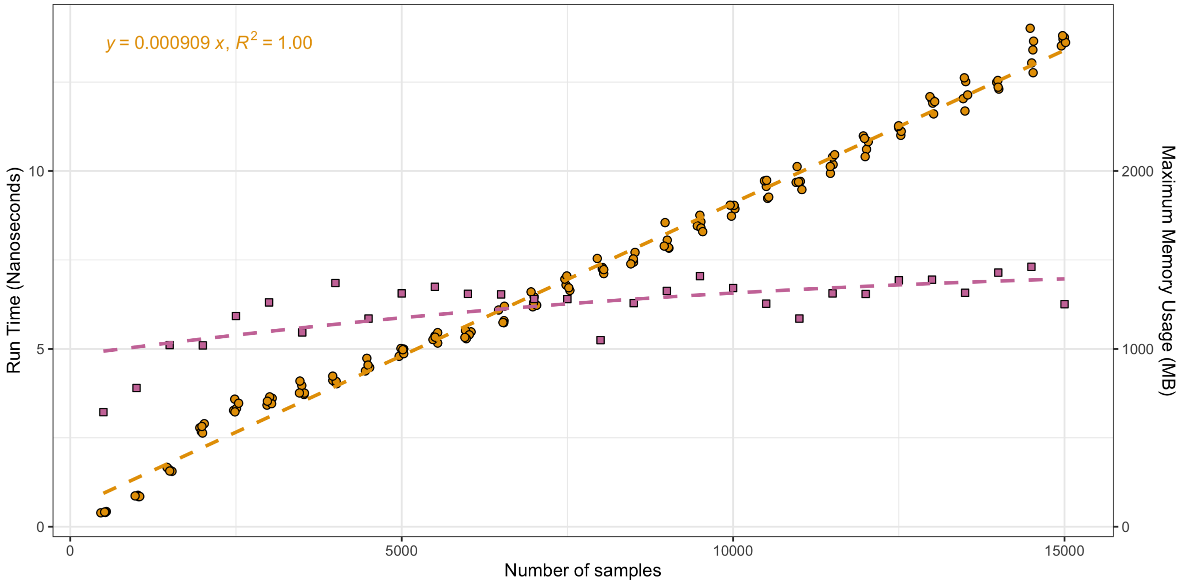

Supplementary Figure 4: Scatter plot of the effect of the number of samples (x-axis) on the total runtime (orange, left y-axis) and maximum memory required (purple, right y-axis). The equation for the regression of the number of samples on the run time is given in the top right corner.

References

R Core Team. *R: A Language and Environment for Statistical Computing*. R Foundation for Statistical Computing, Vienna, Austria, 2025. URL `https://www.R-project.org/`.

Max Kuhn. Building predictive models in R using the caret package. *Journal of statistical software*, 28:1–26, 2008.

Hadley Wickham. *ggplot2: Elegant Graphics for Data Analysis*. Springer-Verlag New York, 2016. ISBN 978-3-319-24277-4. URL `https://ggplot2.tidyverse.org`.

Cara T Mai, James E Kucik, Jennifer Isenburg, Marcia L Feldkamp, Lisa K Marengo, Erin M Bugenske, Phoebe G Thorpe, Jodi M Jackson, Adolfo Correa, Russel Rickard, et al. Selected birth defects data from population-based birth defects surveillance programs in the United States, 2006 to 2010: featuring trisomy conditions. *Birth defects research. Part A, Clinical and molecular teratology*, 97(11):709, 2013.

David Skuse, Frida Printzlau, and Jeanne Wolstencroft. Sex chromosome aneuploidies. *Handbook of clinical neurology*, 147:355–376, 2018.

Fengqing Chao, Patrick Gerland, Alex R Cook, and Leontine Alkema. Systematic assessment of the sex ratio at birth for all countries and estimation of national imbalances and regional reference levels. *Proceedings of the National Academy of Sciences*, 116(19):9303–9311, 2019.

Luca Scrucca, Chris Fraley, T. Brendan Murphy, and Adrian E. Raftery. *Model-Based Clustering, Classification, and Density Estimation Using mclust in R*. Chapman and Hall/CRC, 2023. ISBN 978-1032234953. doi:10.1201/9781003277965. URL `https://mclust-org.github.io/book/`.

James A Fellows Yates, Franziska Aron, Gunnar U Neumann, Irina Velsko, Eirini Skourtanioti, Eleftheria Orfanou, Zandra Fagernas, et al. A–z of ancient dna protocols for shotgun illumina next generation sequencing, 2020.

Heng Li. Aligning sequence reads, clone sequences and assembly contigs with BWA-MEM. *arXiv preprint arXiv:1303.3997*, 2013.

Gabriel Renaud, Udo Stenzel, and Janet Kelso. leeHom: adaptor trimming and merging for Illumina sequencing reads. *Nucleic acids research*, 42(18):e141–e141, 2014.

Selina Carlhoff, Wibhu Kutanan, Adam B Rohrlach, Cosimo Posth, Mark Stoneking, Kathrin Nägele, Rasmi Shoocongdej, and Johannes Krause. Genomic portrait and relatedness patterns of the Iron Age Log Coffin culture in northwestern Thailand. *Nature Communications*, 14(1):8527, 2023.

Lara M Cassidy, Ros Ó Maoldúin, Thomas Kador, Ann Lynch, Carleton Jones, Peter C Woodman, Eileen Murphy, Greer Ramsey, Marion Dowd, Alice Noonan, et al. A dynastic elite in monumental Neolithic society. *Nature*, 582 (7812):384–388, 2020.

Sandra Penske, Adam B Rohrlach, Ainash Childebayeva, Guido Gnecchi-Ruscone, Clemens Schmid, Maria A Spyrou, Gunnar U Neumann, Nadezhda Atanassova, Katrin Beutler, Kamen Boyadzhiev, et al. Early contact between late farming and pastoralist societies in southeastern Europe. *Nature*, 620(7973):358–365, 2023.

Maïté Rivollat, Adam Benjamin Rohrlach, Harald Ringbauer, Ainash Childebayeva, Fanny Mendisco, Rodrigo Barquera, András Szolek, Mélie Le Roy, Heidi Colleran, Jonathan Tuke, et al. Extensive pedigrees reveal the social organization of a Neolithic community. *Nature*, 620(7974):600–606, 2023.

Vanessa Villalba-Mouco, Camila Oliart, Cristina Rihuete-Herrada, Ainash Childebayeva, Adam B Rohrlach, María Inés Fregeiro, Eva Celdrán Beltrán, Carlos Velasco-Felipe, Franziska Aron, Marie Himmel, et al. Genomic transformation and social organization during the Copper Age-Bronze Age transition in southern Iberia. *Science advances*, 7(47): eabi7038, 2021.

Olaf Mersmann. *microbenchmark: Accurate Timing Functions*, 2024. URL `https://CRAN.R-project.org/package=microbenchmark`. R package version 1.5.0.

TV Elzhov, KM Mullen, AN Spiess, B Bolker, and MKM Mullen. Interface to the levenberg-marquardt nonlinear least-squares algorithm found in minpack, plus support for bounds. r package version 1.2-1, 2016.
